# Prognostic Significance of T‐Wave Amplitude Variability for Adverse Cardiovascular Outcomes: A Systematic Review and Meta‐Analysis

**DOI:** 10.1002/joa3.70411

**Published:** 2026-07-02

**Authors:** Yoshihiro Sobue, Kazuya Takeda, Mari Amino, Koichiro Yoshioka, Tomohide Ichikawa, Eiichi Watanabe

**Affiliations:** ^1^ Division of Cardiology, Department of Internal Medicine Fujita Health University Bantane Hospital Nagoya Japan; ^2^ Graduate School of Health Sciences Fujita Health University Graduate School Toyoake Japan; ^3^ Department of Cardiovascular Medicine Tokai University Kanagawa Japan; ^4^ Department of Cardiology Matsumoto Kyoritsu Hospital Matsumoto Japan

**Keywords:** beat‐to‐beat variability, electrocardiography, repolarization instability, risk stratification, sudden cardiac death, ventricular arrhythmias

## Abstract

**Background:**

T‐wave amplitude variability (TAV), a marker of beat‐to‐beat ventricular repolarization instability, has been proposed as a potential risk marker for adverse cardiovascular outcomes, but its prognostic value remains unclear.

**Objectives:**

This meta‐analysis aimed to quantitatively synthesize evidence on the association between elevated TAV and adverse cardiovascular outcomes.

**Methods:**

A systematic literature search of PubMed, Embase, and the Cochrane Library was conducted. Observational studies reporting adjusted effect estimates for the association between TAV and adverse cardiovascular outcomes were included, and separate meta‐analyzes were conducted according to effect measures (hazard ratios [HRs] or odds ratios [ORs]) and exposure definition. Effect estimates were pooled using a random‐effects model. Statistical heterogeneity was assessed using the Q statistic and *I* ^2^.

**Results:**

Seven studies involving 1366 patients were included. HR‐based analyzes (*n* = 3) showed that elevated TAV was significantly associated with an increased risk of adverse outcomes, including mortality and ventricular tachyarrhythmias (pooled HR 2.51, 95% CI 1.57–4.00; *I* ^2^ = 7%). In contrast, OR‐based analyzes showed no statistically significant association between TAV and ventricular tachyarrhythmias, whether evaluated as categorical (high vs. low TAV: pooled OR 3.82, 95% CI 0.87–16.84; *I* ^2^ = 75%) or continuous variable (per 1–μV increase: pooled OR 1.11, 95% CI 0.94–1.30; *I* ^2^ = 70%).

**Conclusion:**

Elevated TAV is associated with an increased risk of adverse cardiovascular outcomes in HR‐based analyzes, supporting its potential utility as a marker of repolarization instability for longitudinal risk stratification. In contrast, its association with ventricular tachyarrhythmias was not significant in OR‐based analyzes, which showed substantial heterogeneity.

**Trial Registration:** PROSPERO: CRD420251171782.

## Graphical abstract

In this systematic review and meta‐analysis of seven studies involving 1366 patients, elevated T‐wave amplitude variability (TAV) was associated with a 2.5‐fold higher risk of adverse cardiovascular outcomes. These findings support TAV as a marker of ventricular repolarization instability for long‐term risk stratification.

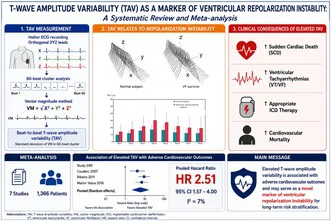

## 1 Introduction

**FIGURE 1:**
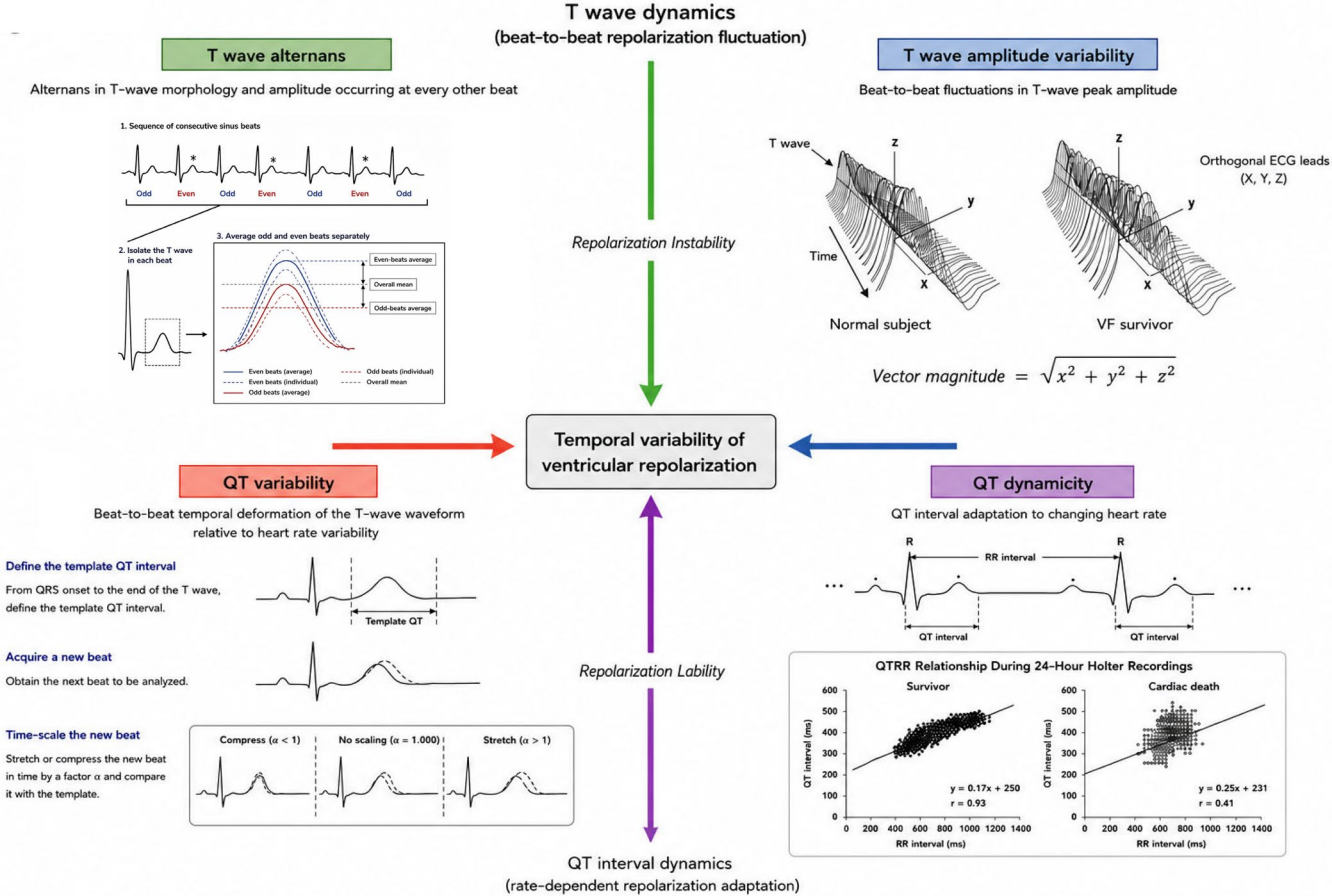
Conceptual framework of repolarization dynamics integrating T‐wave and QT‐interval variability. This schematic summarizes the principal electrocardiographic indices used to characterize the dynamic properties of ventricular repolarization, organized along two conceptual dimensions: T‐wave dynamics (vertical axis) and QT‐interval dynamics (horizontal axis). The upper half of the figure represents markers of repolarization instability, whereas the lower half represents markers of repolarization lability. T‐wave alternans (TWA) (upper left) reflects alternating beat‐to‐beat changes in T‐wave morphology and amplitude occurring every other beat and is a well‐established marker of repolarization instability [3, 6]. TWA amplitude is quantified as the maximal difference in T‐wave morphology between averaged odd and even beats and is expressed in microvolts (μV). T‐wave amplitude variability (TAV) (upper right) quantifies non‐periodic beat‐to‐beat fluctuations in T‐wave amplitude and has been associated with ventricular arrhythmias, sudden cardiac death, and adverse cardiovascular outcomes [7, 9, 10, 11, 12, 13, 14, 15, 16]. TAV is derived from orthogonal X, Y, and Z leads using the vector magnitude (vector magnitude = √(X^2^ + Y^2^ + Z^2^)). Consecutive sinus beats are grouped into clusters of 60 beats, and beat‐to‐beat variability in T‐wave amplitude is quantified within sequential 50‐ms repolarization windows. The maximum variability observed across the analyzed T‐wave segments (max TAV) is commonly used as a marker of repolarization instability. QT variability (lower left) reflects beat‐to‐beat temporal deformation of the entire T‐wave waveform relative to heart rate variability. The illustrated template‐matching approach estimates QT interval fluctuations using the entire T‐wave morphology rather than relying solely on identification of the T‐wave end. QT variability is commonly summarized by the QT Variability Index (QTVI), which compares normalized beat‐to‐beat QT interval variability with normalized heart rate variability [2]. Higher QTVI values indicate excessive repolarization variability beyond that expected from heart rate fluctuations alone. QT dynamicity (lower right) describes the adaptive relationship between ventricular repolarization and cycle length, typically quantified by the QT/RR relationship obtained from ambulatory ECG recordings. A steeper QT/RR slope reflects exaggerated repolarization dependence on heart rate, whereas a reduced correlation between QT and RR intervals indicates impaired coupling between repolarization and cycle length [8]. Both abnormalities have been associated with adverse cardiovascular outcomes, ventricular arrhythmias, and increased mortality. QT dynamicity therefore provides a measure of rate‐dependent repolarization adaptation that complements beat‐to‐beat variability markers. Together, these complementary indices characterize both beat‐to‐beat and rate‐dependent aspects of ventricular repolarization and provide mechanistic and prognostic insights into arrhythmia susceptibility and cardiovascular risk.

Ventricular repolarization instability plays a pivotal role in the pathogenesis of malignant ventricular arrhythmias and sudden cardiac death and has consequently emerged as a key marker for risk stratification [1, 2, 3, 4, 5]. Conceptually, these repolarization dynamics can be represented within a two‐dimensional framework defined by T‐wave dynamics and QT‐interval dynamics, reflecting the temporal fluctuation and rate‐dependent adaptation of ventricular repolarization, respectively [2, 3, 6, 7, 8]. A schematic representation of this conceptual framework is shown in Figure 1. This framework provides an integrated view linking physiological variability to arrhythmic vulnerability.

Building on these technological developments, T‐wave amplitude variability (TAV) has been proposed as a quantitative index of temporal, beat‐to‐beat fluctuations in ventricular repolarization amplitude, typically derived from Holter electrocardiograms (ECGs) using automated analytical platforms [7, 9, 10, 11, 12, 13, 14, 15, 16]. Unlike T‐wave alternans (TWA) [3, 6], which reflects periodic alternation in polarity or amplitude, TAV captures subtle, nonperiodic fluctuations, offering conceptual simplicity within the broader context of T‐wave dynamics. However, its clinical utility remains uncertain due to inconsistent evidence of its prognostic value. Although several previous studies have applied standardized Holter‐based recordings—particularly using proprietary systems such as SpiderView (Ela Medical, Sorin Group, France)—differences in study populations, sample sizes, and endpoint definitions have limited the generalizability of individual results [7, 9, 10, 11, 12, 13, 14, 15, 16].

To address these inconsistencies, we conducted a systematic review and meta‐analysis to assess the prognostic significance of TAV in various clinical settings, as well as to evaluate heterogeneity between studies, possible publication bias, and methodological quality.

## 2 Methods

### 2.1 Search Strategy and Study Selection

This study was conducted in accordance with the Preferred Reporting Items for Systematic Reviews and Meta‐Analyzes (PRISMA) 2020 statement [17], and was prospectively registered with the International Prospective Register of Systematic Reviews (PROSPERO; registration number CRD420251171782). The search strategy was developed in line with the PRISMA‐S extension for reporting search strategies and detailed in Supporting Informations 1 (PRISMA‐S compliance statement). We systematically searched PubMed, Embase, and the Cochrane Library for articles published from January 2000 through December 2024. Search terms included combinations of the following keywords and controlled vocabulary (MeSH/Emtree): “T‐wave,” “sudden cardiac death,” “heart failure,” “ventricular arrhythmia,” “mortality,” “variation,” “variability,” “amplitude.” Boolean operators OR (within concepts) and AND (across concepts) were used to construct the search queries. Full database‐specific strategies are provided in Supporting Informations 2 (Search Strategy). Additionally, reference lists of all eligible articles were manually screened to identify potentially relevant studies.

Two investigators (K.T. and E.W.) independently reviewed and extracted data from eligible studies, including study characteristics (author, year, population, sample size), definition of TAV, clinical endpoints, adjusted effect estimates with corresponding covariates and duration of follow‐up. When multiple statistical models were presented, the most fully adjusted estimate was used for meta‐analysis. Any discrepancies were resolved by discussion and consensus.

Studies were eligible for inclusion if they met the following criteria: (1) enrolled human participants; (2) assessed TAV using Holter electrocardiography with the SpiderView system; (3) reported outcomes including cardiovascular or all‐cause mortality, sudden cardiac death, ventricular tachyarrhythmias, or appropriate implantable cardioverter‐defibrillator (ICD) therapies; and (4) provided adjusted effect estimates with corresponding 95% confidence intervals (CIs). Only peer‐reviewed studies published in English were considered. Exclusion criteria included animal studies, case reports, case series, conference abstracts without full text availability, clinical protocols, practice guidelines, narrative or systematic reviews, meta‐analyzes, editorials, commentaries, book chapters, in silico or in vitro studies, and duplicate publications. A completed PRISMA 2020 checklist is provided in the Supporting Informations 3 (PRISMA 2020 Checklist). For one study Yoshioka et al. [13], the 95% confidence interval (CI) was not available in the published article but was obtained through personal communication with the corresponding author.

### 2.2 Outcome Definition

The primary outcome of this meta‐analysis was adverse cardiovascular outcomes assessed in time‐to‐event analyzes. These outcomes included all‐cause mortality, sudden cardiac death, and appropriate ICD therapy. The secondary outcome was ventricular tachyarrhythmic events assessed as binary outcomes, including sustained ventricular tachycardia, ventricular fibrillation, polymorphic ventricular tachycardia/ventricular fibrillation, and ventricular fibrillation/asystole events.

### 2.3 Data Extraction and Quality Assessment

The methodological quality of the included observational studies was assessed using the Newcastle–Ottawa Scale, which evaluates three domains: selection of study groups, comparability of groups, and ascertainment of outcomes or exposures [18]. Each study was assigned a maximum of nine points; studies scoring eight points or more were considered to be of high methodological quality. No formal assessment of publication bias was performed using funnel plots or statistical tests because the number of studies included in each meta‐analysis was less than 10, for which such methods are considered unreliable.

### 2.4 Statistical Analysis

Given that hazard ratios (HRs) or odds ratios (ORs) represent distinct effect measures, and that definitions of TAV exposure varied across studies (i.e., categorical high vs. low or continuous per–1 μV increase), separate meta‐analyzes were performed. The effect estimates were logarithmically transformed and pooled using a random‐effects model based on the DerSimonian–Laird method to account for heterogeneity between studies. The primary summary measures were pooled adjusted HRs or ORs with 95% CIs. Statistical heterogeneity was assessed using Cochran's Q statistic and quantified with the *I* ^2^ statistic. Interpretation of *I* ^2^ values followed the guidance of the *Cochrane Handbook for Systematic Reviews of Interventions*: 0%–25% (not important), 25%–50% (moderate), 50%–75% (substantial), and 75%–100% (considerable heterogeneity) [19]. Prespecified subgroup analyzes according to ischemic versus nonischemic populations were planned to explore potential sources of heterogeneity. However, because only seven studies met the eligibility criteria, formal subgroup analyzes were not performed. HRs and ORs were analyzed separately because they represent different effect measures and are not directly combinable within a single pooled analysis. To improve transparency with respect to the adjusted effect estimates used in the meta‐analysis, the covariates included in the multivariate adjusted models of the original studies are summarized in Supporting Informations 4 (Table S1). All statistical analyzes were conducted using R software (version 4.3.1; R Foundation for Statistical Computing, Vienna, Austria) with the *meta* package (version 6.5.0).

## 3 Results

### 3.1 Study Selection

**FIGURE 2:**
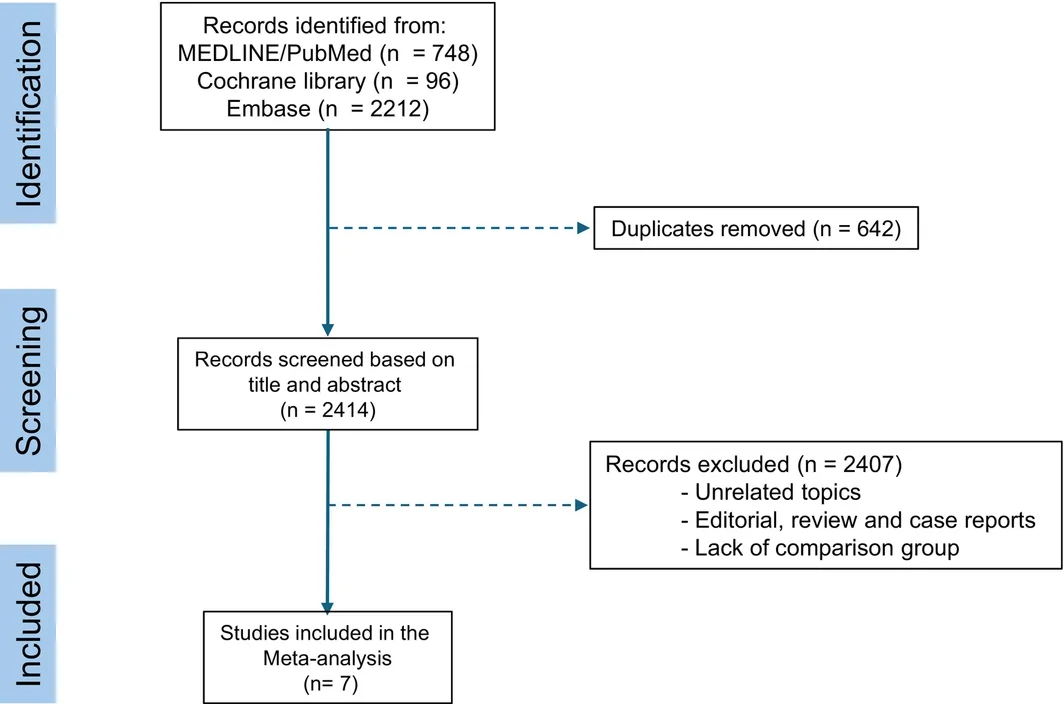
Study selection flow diagram. Flow diagram of study selection according to the PRISMA 2020 guidelines. A total of 3056 records were identified through database searches. After removal of duplicates and screening by title and abstract, seven observational cohort studies were included in the meta‐analysis. PRISMA: Preferred Reporting Items for Systematic Reviews and Meta‐Analyzes.

A total of 3056 records were identified through database searches. After removing 642 duplicates, 2414 records were screened for title and abstract. Of these, 2407 were excluded based on prespecified eligibility criteria. Ultimately, seven observational cohort studies were included in the meta‐analysis (Figure 2).

### 3.2 Study Characteristics

The characteristics of the seven studies are summarized in Table 1. In total, the meta‐analysis included 1366 participants across seven studies. The studies involved heterogeneous patient populations, including individuals with prior myocardial infarction, chronic heart failure, Chagas disease, Brugada syndrome, acute coronary syndrome, and those without structural heart disease. Sample sizes ranged from 43 to 572 participants, with mean ages spanning 41 to 70 years. A detailed summary of covariates included in the adjusted analyzes is provided in Supporting Informations 4 (Table S1).

**TABLE 1:** Baseline characteristics of included studies.

Study, year	Underlying disease/population	Age (mean ± SD)	No. of participants	Primary outcome	Mean follow‐up duration (months)	Exposure definition (High vs. Low/per 1‐μV)	Adjusted effect type (HR/OR)	Cut‐off value (μV)
Couderc et al. [7]	Prior myocardial infarction	70 ± 14	275	Appropriate ICD therapy	24	High versus low	HR	59
Ribeiro et al. [10]	Chagas disease	42 ± 9	113	All‐cause death	106	High versus low	HR	30
Sobue et al. [11]	No structural heart disease	41 ± 16	60	VT/VF	23	High versus low	OR	—
Yoshioka et al. [13]	Brugada syndrome	47 ± 13	127	VF, asystole	—	Per 1‐μV	OR	54
Ichikawa et al. [14]	No structural heart disease	52 ± 17	43	VT/VF	—	High versus low	OR	33
Martin‐Yebra et al. [15]	Chronic heart failure, AF	69 ± 10	176	Sudden cardiac death	48	High versus low	HR	32.33
Makino et al. [16]	Acute coronary syndrome	67 ± 12	572	VT/VF	—	Per 1‐μV	OR	—

*Note:* Data represent mean ± SD.

Abbreviations: HR: hazard ratio, ICD: implantable cardioverter‐defibrillator, OR: odds ratio, VF: ventricular fibrillation, VT: ventricular tachycardia.

### 3.3 Methodological Quality of Included Studies

Methodological quality was evaluated using the Newcastle‐Ottawa scale. As summarized in Table 2, all studies were rated as high quality, with total scores ranging from 8 to 9 points.

**TABLE 2:** Quality assessment of included studies using the Newcastle–Ottawa Scale.

Study	Study design	Selection (Max 4 stars)	Comparability (Max 2 stars)	Outcome/exposure (Max 3 stars)	Total score (Max 9)
Couderc et al. [7]	Cohort	★★★★	★★	★★★	9
Ribeiro et al. [10]	Cohort	★★★★	★★	★★★	9
Sobue et al. [11]	Cohort	★★★★	★★	★★☆	8
Yoshioka et al. [13]	Cohort	★★★★	★★	★★★	9
Ichikawa et al. [14]	Cohort	★★★★	★★	★★☆	8
Martin‐Yebra et al. [15]	Cohort	★★★★	★★	★★★	9
Makino et al. [16]	Cohort	★★★★	★★	★★★	9

### 3.4 Meta‐Analysis of Hazard Ratios

**FIGURE 3:**
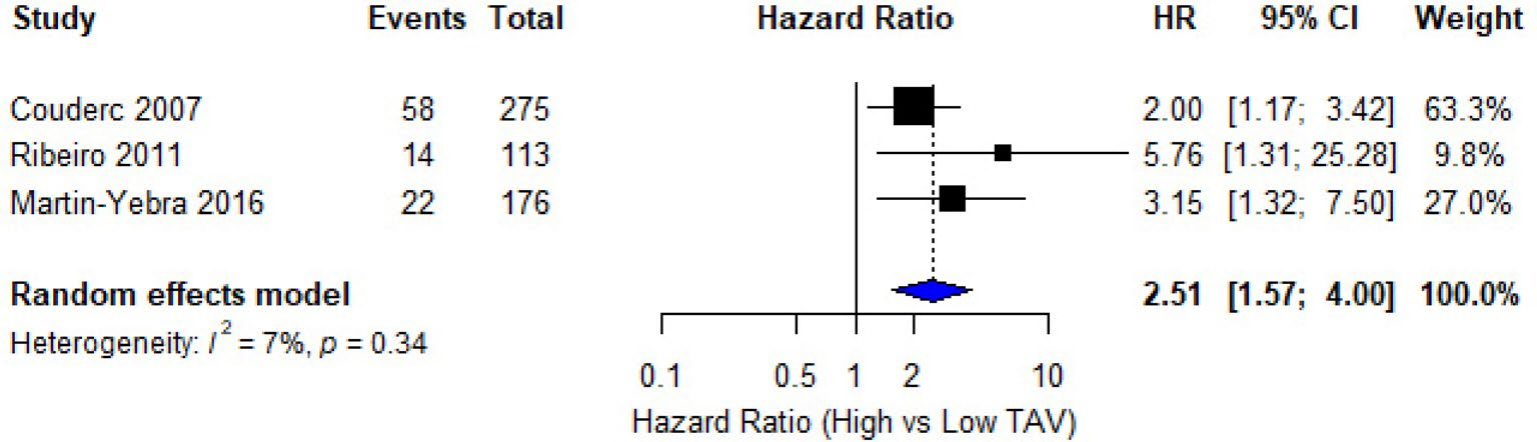
Hazard ratio‐based meta‐analysis of adverse cardiovascular outcomes. Forest plot of HR–based meta‐analysis evaluating the association between elevated TAV and adverse cardiovascular outcomes. Individual study estimates and the pooled HR were calculated using a random‐effects model. Squares indicate study‐specific effect estimates with sizes proportional to study weight; horizontal lines represent 95% CIs; and the diamond indicates the pooled estimate. CI: Confidence interval, HR: Hazard ratio, TAV: T‐wave amplitude variability.

Three studies reporting adjusted HRs were included in a random‐effects meta‐analysis (Figure 3). Elevated TAV was significantly associated with an increased risk of adverse cardiovascular outcomes (pooled HR 2.51, 95% CI 1.57–4.00). The heterogeneity was low (*I* ^2^ = 7%, *p* = 0.34), indicating consistent findings between studies.

### 3.5 Meta‐Analysis of Odds Ratios

**FIGURE 4:**
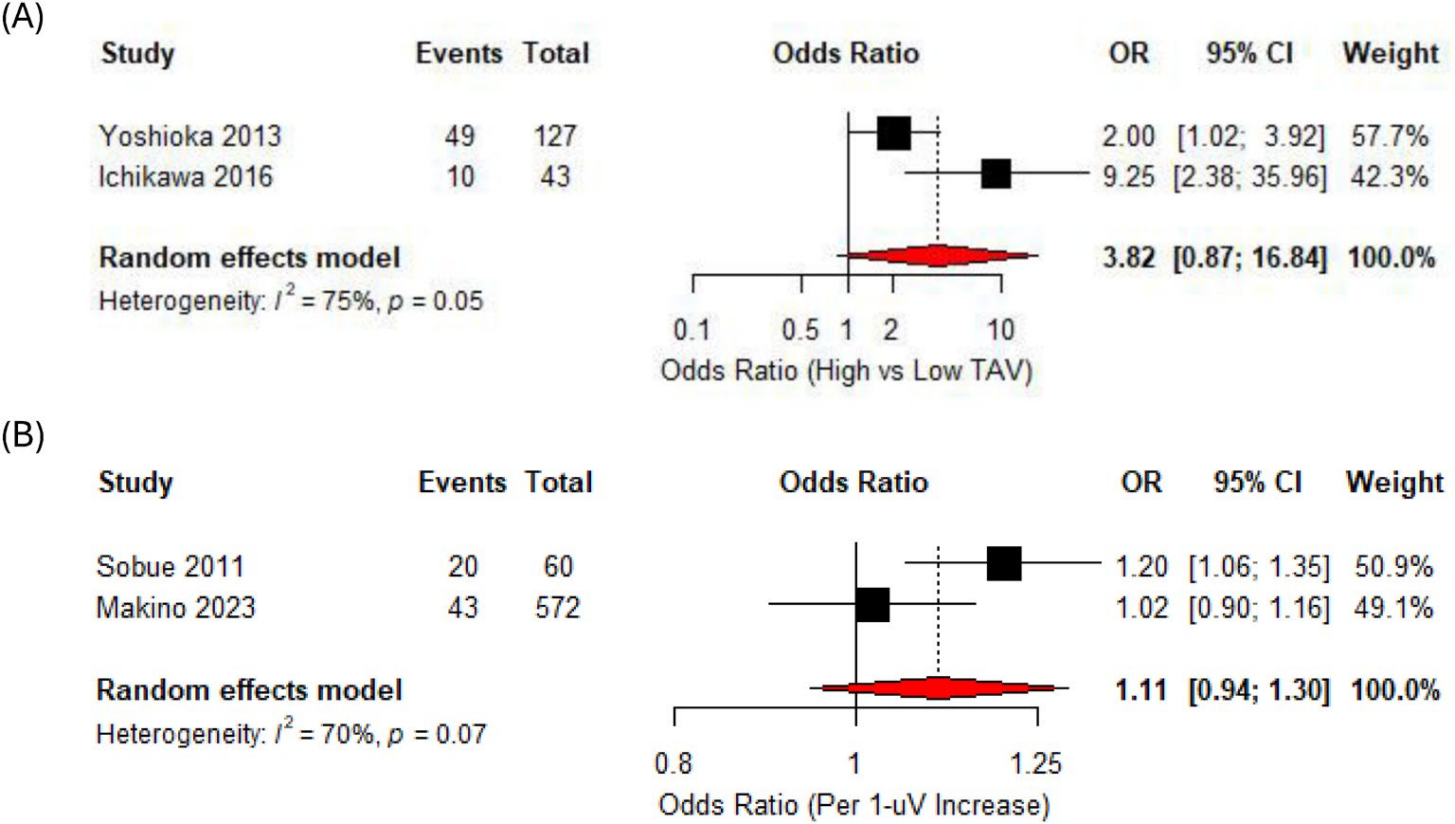
Odds ratio‐based meta‐analyzes of ventricular tachyarrhythmias. Forest plots of OR–based meta‐analyzes evaluating the association between TAV and ventricular tachyarrhythmias. (A) Categorical analysis comparing high versus low TAV. (B) Continuous analysis evaluating the effect per 1–μV increase in TAV. Pooled ORs were calculated using a random‐effects model. Squares indicate study‐specific effect estimates with 95% CIs, and diamonds represent pooled estimates. CI: Confidence interval, OR: Odds ratio, TAV: T‐wave amplitude variability.

Meta‐analyzes based on ORs were performed separately due to differences in outcome definitions and exposure modeling. In categorical analyzes comparing high versus low TAV (Figure 4A), the pooled OR indicated an increased risk of ventricular tachyarrhythmias, although the association was not statistically significant (pooled OR 3.82, 95% CI 0.87–16.84). Substantial heterogeneity was noted (*I* ^2^ = 75%, *p* = 0.05). Similarly, in continuous analyzes evaluating the effect per 1–μV increase in TAV (Figure 4B), no significant association with ventricular tachyarrhythmias was observed (pooled OR 1.11, 95% CI 0.94–1.30), with moderate‐to‐high heterogeneity (*I* ^2^ = 70%, *p* = 0.07). Despite variations in effect measures and exposure definitions, the direction of effect estimates in all analyzes consistently suggested a positive association between elevated TAV and adverse cardiovascular outcomes.

## 4 Discussion

### 4.1 Major Findings

In this first meta‐analysis of Holter‐derived TAV, which included more than 1300 subjects, elevated TAV was significantly associated with an increased risk of adverse cardiovascular outcomes in HR‐based, time‐to‐event analyzes. In contrast, its association with ventricular tachyarrhythmias was inconclusive in OR‐based analyzes, although the direction of effect estimates consistently favored higher risk with increasing TAV. These findings suggest that TAV reflects ventricular repolarization vulnerability that is more closely related to long‐term cardiovascular risk. The divergent results observed between analytical approaches underscore the need for cautious interpretation and highlight the importance of the methodological context in evaluating repolarization markers.

### 4.2 Comparison With Previous Studies and Other ECG Markers

Temporal variability of ventricular repolarization has been evaluated using long‐term electrocardiographic recordings, which allow the evaluation of dynamic fluctuations under physiological conditions. Previous Holter‐based approaches include analyzes of QT dynamicity [2], circadian variation of heart rate–corrected QT interval (QTc), measures of QT interval variability [8], and ambulatory electrocardiographic TWA [20]. Among these, ambulatory ECG–derived TWA has been shown to predict fatal cardiac events, including sudden cardiac death, in selected populations [20]. In this context, TAV offers an alternative Holter‐based metric that captures beat‐to‐beat, nonperiodic fluctuations in ventricular repolarization. The current meta‐analysis demonstrates that, despite the heterogeneity of study populations, analytic methods, and outcome definitions, elevated TAV consistently predicts adverse cardiovascular outcomes, particularly when assessed using time‐to‐event methodologies.

Although only one included study focused exclusively on patients with atrial fibrillation, the finding that a comparable TAV cut‐off value retained prognostic value despite significant variability of the RR interval and fibrillatory wave interference is clinically notable [15]. This observation suggests that TAV may serve as a robust marker of repolarization instability even in the setting of atrial fibrillation, which warrants further investigation.

### 4.3 Methodological Considerations and Standardization of TAV Assessment

An important consideration when interpreting the current evidence is the lack of methodological standardization in the assessment of TAV. Although all included studies quantified the beat‐to‐beat variability of ventricular repolarization using Holter‐derived T‐wave amplitude analysis, there were substantial differences in recording protocols, signal‐processing approaches, exposure definitions, and analytical endpoints.

All studies employed the SpiderView/SyneTVar platform and assessed TAV using clusters of 60 consecutive sinus beats. However, the recording conditions varied considerably, ranging from short resting recordings and nocturnal recordings to full 24‐h Holter monitoring. Furthermore, different studies summarized within‐patient variability using different approaches, including maximum TAV, mean TAV, median TAV, or modified indices such as the index of T‐wave variation developed for atrial fibrillation populations. Consequently, there is currently no consensus on the most informative patient‐level summary metric at the patient level. Threshold values that define elevated TAV also varied substantially between studies, ranging from approximately 20 to 60 μV, while some studies expressed repolarization variability as variance‐based metrics (μV^2^). These methodological differences likely contributed to the heterogeneity observed between studies and currently limit the establishment of a universally applicable clinical cut‐off value. However, an important observation is that the overall direction of association remained remarkably consistent despite these methodological differences. Elevated TAV was generally associated with a higher risk of adverse cardiovascular outcomes in studies employing different durations of recording, analytical algorithms, patient populations and outcome definitions.

Future investigations should focus on standardizing TAV acquisition and analysis, determining the optimal approach to summarize patient variability, establishing reference values in different populations, and clarifying whether maximum, mean, median, or alternative variability measures provide the greatest prognostic value. Such efforts will be essential before TAV can be incorporated into routine clinical risk stratification.

### 4.4 Incremental Value of the Present Meta‐Analysis

Importantly, the present study expands the existing literature on repolarization markers in several ways. Previous investigations of repolarization instability have focused primarily on QT variability, QT dynamicity, and TWA, while evidence regarding TAV has remained fragmented and limited to relatively small observational studies. To our knowledge, this is the first systematic review and meta‐analysis that specifically evaluates the prognostic significance of Holter‐derived TAV in various clinical settings. By quantitatively synthesizing the available evidence, the present study demonstrates a consistent association between elevated TAV and adverse cardiovascular outcomes in time‐to‐event analyzes despite substantial differences in patient populations and study designs. These findings support TAV as a potentially clinically relevant marker of repolarization instability and provide a framework for future prospective validation studies and methodological standardization.

### 4.5 Pathophysiological and Analytical Interpretation of TAV

The electrophysiological mechanisms underlying beat‐to‐beat TAV remain incompletely understood. Conceptually, TAV is believed to reflect temporal lability in ventricular repolarization, arising from dynamic interactions among regional heterogeneity in action potential duration, autonomic modulation, and transient ischemic or metabolic influences. However, direct experimental evidence linking TAV to specific cellular or tissue‐level mechanisms is limited.

TAV has been discussed as a related but distinct phenomenon from TWA. Whereas TWA represents periodic, beat‐to‐beat alternation typically emerging under conditions of marked electrical instability, TAV captures nonperiodic, stochastic fluctuations in repolarization. As such, TAV may represent a broader and more continuously present substrate vulnerability rather than discrete arrhythmia‐triggering events. Whether TAV and TWA serve as independent, overlapping, or complementary markers of repolarization instability remains an open question [21, 22]. These conceptual distinctions may help explain the discrepant findings observed in the current meta‐analysis. Elevated TAV was consistently associated with adverse cardiovascular outcomes in HR–based, time‐to‐event analyzes, but not with ventricular tachyarrhythmias in OR–based analyzes. HRs incorporate temporal dimensions of risk and are therefore better suited to capture chronic vulnerability, which TAV may reflect. On the contrary, ORs evaluate binary outcomes at fixed time points, which may underrepresent cumulative or latent risk. Furthermore, although ventricular tachyarrhythmia was uniformly defined across studies, OR‐based analyzes were characterized by substantial heterogeneity in population characteristics and exposure modeling (e.g., dichotomous vs. continuous TAV), potentially attenuating effect estimates. Ventricular tachyarrhythmias themselves are episodic events often triggered by transient stimuli, whereas HR‐based outcomes such as mortality or appropriate ICD therapy may better reflect persistent electrophysiological substrate abnormalities captured by TAV. Taken together, these considerations suggest that TAV is a marker of long‐term repolarization instability and may be more appropriately evaluated using time‐to‐event analytical frameworks. More mechanistic and prospective studies are warranted to elucidate the physiological basis of TAV and to clarify its prognostic role in relation to other repolarization‐based markers [4].

### 4.6 Study Limitations

Several limitations should be acknowledged. First, all included studies were observational in design, and therefore causality cannot be inferred from the observed associations. Second, the number of eligible studies was relatively small, limiting statistical power and restricting detailed exploration of heterogeneity. Third, substantial heterogeneity was observed in the OR‐based analyzes, which may reflect differences in study populations, underlying disease substrates, endpoint definitions, and exposure modeling strategies. Fourth, the definition of elevated TAV was not standardized, and threshold values varied considerably between studies, limiting direct clinical translation. Fifth, although all studies employed Holter‐derived TAV assessment, differences in recording conditions, analytical algorithms, and study‐specific methodologies may have influenced the reported effect estimates. Finally, publication bias could not be formally assessed because fewer than 10 studies were available for each meta‐analysis.

## 5 Conclusions

This systematic review and meta‐analysis suggest that elevated TAV is significantly associated with adverse cardiovascular outcomes in HR‐based analyzes. In contrast, its association with ventricular tachyarrhythmias was not statistically significant in ORs ratio–based analyzes, which exhibited substantial heterogeneity. These findings support TAV as a marker of ventricular repolarization vulnerability with potential utility for longitudinal risk stratification. However, the evidence does not currently support its use to predict discrete arrhythmic events, guide ICD implantation, or establish universal clinical cutoff values. Further large‐scale, prospective studies using standardized TAV definitions and harmonized analytical frameworks are warranted to clarify its prognostic value and clinical applicability.

## Author Contributions

E.W. and Y.S. conceived the study and drafted the manuscript. K.T. and E.W. performed the literature search and data extraction. K.T. conducted the statistical analysis. All authors contributed to manuscript drafting and approved the final version.

## Funding

The authors have nothing to report.

## Ethics Statement

Ethical approval was not required for this study because it was a systematic review and meta‐analysis of previously published data.

## Consent

Informed consent was not required for this study.

## Conflicts of Interest

Dr. Eiichi Watanabe serves as a consultant to Fukuda Denshi. The other authors declare no conflicts of interest.

## Supporting information


**Supporting Informations 1** PRISMA‐S compliance statement.
Supporting Informations 2. Search Strategy (PRISMA‐S compliant).
Supporting Informations 3. PRISMA 2020 checklist.
**Table S1:** Covariates included in adjusted analyzes of the studies included in the meta‐analysis.

## Data Availability

The data that support the findings of this study are available on request from the corresponding author. The data are not publicly available due to privacy or ethical restrictions.
