# Correction to “R1 Prognostic Significance of T‐Wave Amplitude Variability for Adverse Cardiovascular Outcomes: A Systematic Review and Meta‐Analysis”

**DOI:** 10.1002/joa3.70493

**Published:** 2026-10-06

Y. Sobue , K. Takeda , M. Amino , K. Yoshioka , T. Ichikawa , and E. Watanabe , “R1 Prognostic Significance of T‐Wave Amplitude Variability for Adverse Cardiovascular Outcomes: A Systematic Review and Meta‐Analysis,” Journal of Arrhythmia 42, no. 4 (2026): e70411, 10.1002/joa3.70411.PMC1332481142394818

The title of the article “R1 Prognostic Significance of T‐Wave Amplitude Variability for Adverse Cardiovascular Outcomes: A Systematic Review and Meta‐Analysis” is corrected to “Prognostic Significance of T‐Wave Amplitude Variability for Adverse Cardiovascular Outcomes: A Systematic Review and Meta‐Analysis”.

The online version of the article is corrected accordingly.

We apologize for this error.

