# Supplementary material for: R1 Prognostic Significance of T‐Wave Amplitude Variability for Adverse Cardiovascular Outcomes: A Systematic Review and Meta‐Analysis
Source: J Arrhythm. 2026 Jul 2;42(4):e70411. doi: 10.1002/joa3.70411 (PMC13324811; doi:10.1002/joa3.70411)
Supplement: Supplementary file 1 — Supporting Informations 1 PRISMA‐S compliance statement. Supporting Informations 2. Search Strategy (PRISMA‐S compliant). Supporting Informations 3. PRISMA 2020 checklist. Table S1: Covariates included in adjusted analyzes of the studies included in the meta‐analysis. [file JOA3-42-e70411-s001.docx]

**Supplementary Materials**

**Supplementary Materials 1. PRISMA-S compliance statement**

The complete electronic search strategies for all databases, including controlled vocabulary terms and free-text keywords, search fields, limits, and execution dates, are reported in this Supplementary Methods section in accordance with **PRISMA-S guidelines**.

| **PRISMA-S Item** | **Checklist item** | **Reported in this study** |
| --- | --- | --- |
| **1** | Name of the database(s) searched | PubMed, Embase, and Cochrane Library (CENTRAL) |
| **2** | Date(s) each database was searched | From inception to April 30, 2024 |
| **3** | Platform(s) used (e.g., PubMed, embase.com) | PubMed (NLM), Embase (Elsevier), Cochrane Library |
| **4** | Full search strategy for each database | Reported in Supplementary Methods – Search Strategy |
| **5** | Use of controlled vocabulary (e.g., MeSH, Emtree) | Yes (MeSH in PubMed; Emtree in Embase) |
| **6** | Use of free-text terms | Yes (Title/Abstract keywords) |
| **7** | Boolean operators used (AND, OR) | OR used within concepts; AND used between concepts |
| **8** | Limits applied (e.g., humans, language) | Humans and English language |
| **9** | Search fields used (e.g., title, abstract) | Title/Abstract and controlled vocabulary fields |
| **10** | Search filters used | None (no date or study-design filters applied) |
| **11** | Rationale for search limits | To focus on human clinical studies and ensure interpretability |
| **12** | Deduplication process | Duplicates removed prior to screening |
| **13** | Citation searching (reference lists) | Manual screening of reference lists performed |
| **14** | Use of automation tools | None |
| **15** | Search update | Not applicable (single comprehensive search) |
| **16** | Deviations from protocol | None |
| **17** | Availability of search strategies | Provided in Supplementary Methods |
| **18** | Reporting standard followed | PRISMA 2020 and PRISMA-S |

**Supplementary Materials** **2. Search Strategy (PRISMA-S compliant)**

**Databases and search dates**

A systematic literature search was conducted in **PubMed**, **Embase**, and the **Cochrane Library (CENTRAL)** from database inception to **April 30, 2024**.

**Search concepts**

The search strategy was structured around two key concepts:

1. **Exposure:** T-wave amplitude variability and related terms
2. **Outcomes:** Ventricular arrhythmias, sudden cardiac death, mortality, and heart failure

Synonymous terms within each concept were combined using the **OR** operator, and different concepts were combined using **AND**.

**PubMed (MEDLINE)**

**Search fields:** MeSH Terms and Title/Abstract
**Limits:** Humans; English language

(

("T-Wave"[MeSH] OR "Ventricular Repolarization"[MeSH]

OR t-wave[tiab] OR "t wave"[tiab] OR twave[tiab])

AND

(amplitude[tiab] OR variability[tiab] OR variation[tiab]

OR "beat-to-beat"[tiab] OR "beat to beat"[tiab])

)

AND

(

"Ventricular Arrhythmias"[MeSH]

OR "Sudden Cardiac Death"[MeSH]

OR "Mortality"[MeSH]

OR "Heart Failure"[MeSH]

OR arrhythmia*[tiab]

OR "ventricular arrhythmia*"[tiab]

OR "sudden cardiac death"[tiab]

OR mortality[tiab]

OR death[tiab]

OR "heart failure"[tiab]

)

**Embase (Elsevier)**

**Search fields:** Emtree terms and Title/Abstract
**Limits:** Humans; English language (applied using database filters)

(

('t wave'/exp OR 'ventricular repolarization'/exp

OR t-wave:ti,ab OR 't wave':ti,ab OR twave:ti,ab)

AND

(amplitude:ti,ab OR variability:ti,ab OR variation:ti,ab

OR 'beat-to-beat':ti,ab OR 'beat to beat':ti,ab)

)

AND

(

'ventricular arrhythmia'/exp

OR 'sudden cardiac death'/exp

OR 'mortality'/exp

OR 'heart failure'/exp

OR arrhythmia*:ti,ab

OR 'ventricular arrhythmia*':ti,ab

OR 'sudden cardiac death':ti,ab

OR mortality:ti,ab

OR death:ti,ab

OR 'heart failure':ti,ab

)

**Cochrane Library (CENTRAL)**

**Search fields:** Title, Abstract, Keywords
**Limits:** Trials (CENTRAL)

(

(t-wave OR "t wave" OR twave)

AND

(amplitude OR variability OR variation OR "beat-to-beat" OR "beat to beat")

)

AND

(

"ventricular arrhythmia*"

OR arrhythmia*

OR "sudden cardiac death"

OR mortality

OR death

OR "heart failure"

)

**Additional search methods**

To ensure comprehensive coverage, the reference lists of all included studies and relevant review articles were **manually screened** for additional eligible publications. No further studies meeting the inclusion criteria were identified through manual searching.

**Supplementary Materials 3. PRISMA 2020 Checklist**

| **Section** | **Item No.** | **PRISMA 2020 Checklist Item** | **Location in Manuscript** |
| --- | --- | --- | --- |
| **TITLE** | 1 | Identify the report as a systematic review, meta-analysis, or both | Title page |
| **ABSTRACT** | 2 | Provide a structured summary including objectives, data sources, eligibility criteria, synthesis methods, results, limitations, conclusions, and registration number | Abstract |
| **INTRODUCTION** | 3 | Describe the rationale for the review in the context of existing knowledge | Introduction |
|  | 4 | Provide an explicit statement of the objectives or questions being addressed | Introduction (final paragraph) |
| **METHODS** | 5 | Specify whether a review protocol exists and provide registration information | Methods – Search strategy and study selection |
|  | 6 | Specify eligibility criteria and rationale | Methods – Search strategy and study selection |
|  | 7 | Describe all information sources and date last searched | Methods – Search strategy and study selection |
|  | 8 | Present full search strategy for at least one database | Supplementary Methods |
|  | 9 | Describe the process for study selection | Methods – Search strategy and study selection |
|  | 10 | Describe the data collection process | Methods – Search strategy and study selection |
|  | 11 | List and define all data items | Methods – Search strategy and study selection |
|  | 12 | Describe methods used for assessing risk of bias in individual studies | Methods – Data extraction and quality assessment |
|  | 13 | State the principal summary measures | Methods – Statistical analysis |
|  | 14 | Describe methods of synthesis and measures of consistency | Methods – Statistical analysis |
|  | 15 | Describe methods for assessing risk of bias across studies | Methods – Data extraction and quality assessment |
|  | 16 | Describe additional analyses (e.g., subgroup analyses) | Methods – Statistical analysis |
| **RESULTS** | 17 | Give numbers of studies screened, assessed, and included, with reasons for exclusions | Results – Study selection; Figure 1 |
|  | 18 | Present characteristics of included studies | Results – Study characteristics; Table 1 |
|  | 19 | Present risk of bias within studies | Results – Methodological quality; Table 2 |
|  | 20 | Present results of individual studies | Results – Meta-analysis sections; Figures 2–4 |
|  | 21 | Present results of each synthesis | Results – Meta-analysis sections |
|  | 22 | Present assessment of risk of bias across studies | Results – Meta-analysis sections |
|  | 23 | Present results of additional analyses | No additional analyzes were performed because of the limited number of eligible studies. |
| **DISCUSSION** | 24 | Summarize main findings and strength of evidence | Discussion – Major findings |
|  | 25 | Discuss limitations of the evidence | Discussion – Study limitations |
|  | 26 | Provide a general interpretation and implications | Discussion – Conclusions |
| **OTHER INFORMATION** | 27 | Describe sources of funding and role of funders | Funding / Acknowledgment |

**Supplementary Materials 4.**

**Table S1. Covariates included in adjusted analyses of the studies included in the meta-analysis**

| **Study** | **Outcome** | **Effect measure** | **Adjustment variables included in final model** |
| --- | --- | --- | --- |
| Couderc et al., 2007 | Appropriate ICD therapy for VT/VF | HR | NYHA class, heart rate, LVEF, BUN, sex, RMSSD, SDNN, HF, LF |
| Ribeiro et al., 2011 | All-cause mortality | HR | QRS duration >133 ms, LVEF <50%, VT during stress testing or Holter monitoring |
| Martin-Yebra et al., 2016 | Sudden cardiac death | HR | Age, sex, NYHA class, LVEF ≤35%, diabetes; additional model including beta-blocker therapy, digoxin, and amiodarone use |
| Sobue et al., 2011 | VT/VF occurrence | OR | Age, sex, beta-blocker therapy |
| Yoshioka et al., 2013 | VF/asystole events | OR | Not reported (receiver operating characteristic curve and discriminant analyses were performed, but multivariable covariates were not explicitly described) |
| Ichikawa et al., 2016 | VT/VF | OR | Age, sex |
| Makino et al., 2023 | Acute-phase VT/VF after ACS | OR | Age, sex, Killip class, LVEF |

The table summarizes the covariates included in the adjusted statistical models reported by each original study. The variables retained in the final model may differ according to study-specific model selection procedures. ACS, acute coronary syndrome; BUN, blood urea nitrogen; HF, high-frequency power; HR, hazard ratio; ICD, implantable cardioverter-defibrillator; LF, low-frequency power; LVEF, left ventricular ejection fraction; NYHA, New York Heart Association functional class; OR, odds ratio; RMSSD, root mean square of successive differences; SDNN, standard deviation of normal-to-normal intervals; VF, ventricular fibrillation; VT, ventricular tachycardia.
